# Prevalence and Risk Factors of Peri-Implant Disease: A Retrospective Case-Control Study in Western China

**DOI:** 10.3390/ijerph191912667

**Published:** 2022-10-03

**Authors:** Rui Zhao, Wen Zhao, Jin Huang, Ming Fang, Yan Dong, Jihua Chen, Zhaohua Ji, Min Tian

**Affiliations:** 1 State Key Laboratory of Military Stomatology, School of Stomatology, The Fourth Military Medical University, Xi’an 710032, China; 2 National Clinical Research Center for Oral Diseases, School of Stomatology, The Fourth Military Medical University, Xi’an 710032, China; 3 Shaanxi Key Laboratory of Stomatology, School of Stomatology, The Fourth Military Medical University, Xi’an 710032, China; 4 Department of Prosthodontics, School of Stomatology, The Fourth Military Medical University, Xi’an 710032, China; 5 Department of Epidemiology, School of Public Health, Air Force Medical University, Xi’an 710032, China; 6 Ministry of Education Key Lab of Hazard Assessment and Control in Special Operational Environment, Air Force Medical University, Xi’an 710032, China

**Keywords:** dental implants, peri-implantitis, peri-implant mucositis, prevalence, risk factor

## Abstract

Background: The present study aimed to investigate the prevalence of peri-implant disease and identify potential disease risk factors in western China. Methods: The present retrospective study was conducted in 131 consecutive patients receiving 248 dental implants treated with implant-supported prostheses with a mean follow-up of 2.52 years. Several patient-related, implant-related, and oral hygiene maintenance factors were analyzed. Results: Peri-implant disease developed in 68 (51.91%) patients and 110 (44.35%) implants. The prevalence of peri-implant mucositis and peri-implantitis were 45.80% and 7.63%, respectively, at the subject level, and 36.69% and 7.66%, respectively, at the implant level. Multivariate analysis exhibited that male [odds ratio (OR) = 1.91; 95% confidence interval (CI): 1.02–3.57; *p* = 0.04], implant length < 10mm (OR = 7.87; 95% CI:1.62–38.46; *p* = 0.01), poor proximal contact of the prosthesis (OR = 1.90; 95% CI: 1.06–3.42; *p* = 0.03), tooth brushing once a day (OR = 3.11; 95% CI: 1.26–7.68; *p* = 0.04) and moderate periodontitis (OR = 13.00; 95% CI: 4.38–38.60; *p* < 0.01) were independent risk factors for peri-implant disease.

## 1. Introduction

Dental implants were an effective method for replacing missing teeth in partially edentulous patients. However, peri-implant connective tissue inflammation with or without progressive loss of supporting bone, known as peri-implant mucositis and peri-implantitis [1], may be associated with this treatment. According to the peri-implant diseases and conditions classification developed at the 2017 World Workshop on the Classification of Periodontal and Peri-Implant Diseases and Conditions, the diagnostic definition of peri-implant health was based on the absence of peri-implant soft tissue inflammation (redness, swelling, and profuse bleeding on probing) and additional bone loss following initial healing [2]. A meta-analysis by Derks J et al. [3] estimated the weighted mean prevalence of peri-implant mucositis and peri-implantitis at 43% (95% CI: 32–54%) and 22% (95% CI: 14–30%). Changi [4] used a validated reference cohort comprising patients (2127 patients and 6129 implants) receiving dental implants over 3.5 years and exhibited a 34% prevalence of peri-implantitis on the patient level [standard error (SE): 3.1%] and 21% on the implant level (SE: 1.7%). The prevalence of peri-implant disease changed significantly due to the inconsistent peri-implant disease diagnostic criteria, distribution of population and pathogenic factors, and influence of individual factors.

Studies had identified several risk indicators for peri-implant diseases, such as implant design, patient-related factors, and environmental factors, such as periodontitis and smoking. The recent consensus report identified history of periodontitis, poor plaque control and lack of regular maintenance therapy as risk factors for peri-implant disease [5,6,7,8,9,10].

However, few studies had focused on the prevalence and risk factors of peri-implant diseases in China. Therefore, the present longitudinal study sought to evaluate the prevalence of peri-implant disease as well as the impact of several independent implant and patient-related risk factors on the development of peri-implant disease.

## 2. Materials and Methods

### 2.1. Study Design

**Figure 1:**
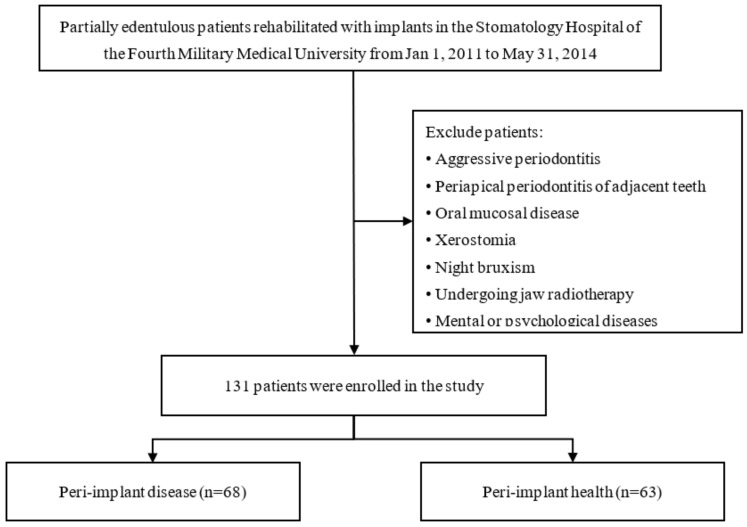
Flow chart of patient selection.

The present longitudinal study was processed after the institutional ethics clearance (approval Number: IRB-Rev-2014027), and conducted by the Helsinki declaration of human studies. All subjects received detailed information about the procedures, risks, and alternatives and were required to sign an informed consent form before participation. The study was registered and approved on Clinicaltrials.gov PRS (https://register.clinicaltrials.gov/ accessed on 14 November 2014) in the United States (approval number: NCT02662361) and adhered to the STROBE guidelines. Partially edentulous individuals with normal jaw relationships who rehabilitated with implants in function for at least one year at the Department of Prosthodontics of the Stomatology Hospital of the Fourth Military Medical University between January 2011 and May 2014 were included in the study. The Exclusion criteria included patients undergoing pre-and post-implantation jaw radiotherapy; those exhibiting night bruxism, xerostomia, oral mucosal disease, or periapical periodontitis of adjacent teeth; patients with mental or psychological diseases affecting treatment response; and those exhibiting aggressive periodontitis [11]. The patient selection flow was shown in Figure 1.

### 2.2. Case and Control Definition

Patients with the peri-implant disease were assigned to the case group, while patients with peri-implant health were assigned to the control group. The applied definitions of peri-implant healthy and diseases were primarily based on the consensus report of the Eighth European Workshop on Periodontolog [12] at the time of study design, peri-implant mucositis was defined as probing depth ≥4 mm, and bleeding on probing and bone loss <2 mm. Peri-implantitis was defined as probing depth >4 mm, bone loss ≥2 mm, and the presence of bleeding on probing or periodontal abscess. Peri-implant mucositis and peri-implantitis were collectively known as peri-implant diseases [13].

### 2.3. Clinical Examination and Quality Control

Patient information was collected and collated including sex, age, systematic medical history, history of smoking and alcohol consumption, causes of tooth loss at the implant site, daily oral maintenance habits, and periodontal maintenance and treatment condition.

Plastic periodontal probe (Hu-Friedy, Chicago, IL, USA) was used for a comprehensive periodontal examination, including modified plaque index (mPLI), periodontal probing depth (PD), and clinical attachment level, and modified sulcus bleeding index (mSBI). Each implant was probed at six sites with a probing force of 0.25 N, recording PD, bleeding, and/or suppurating for per site.

EXpert DC X-ray machine was used to take apical radiography with parallel projection technology. An X-ray positioner was used to ensure that the same patient was photographed at the same angle as before. The amount of bone loss around the implant was measured, and the site with the most severe bone loss was selected as the value of bone loss of the implant.

The clinical examination was conducted by an experienced dentist who has trained and calibrated by professional periodontists before the study. In order to assess the examiner’s credibility during a clinical examination, the key indexes for identifying peri-implant diseases were recorded in a sample of ten randomly selected patients. Probing depth, peri-implant modified plaque index, and probing bleeding index were among them. These indexes were re-measured at different times within 1 day. The result consistency between the two examinations was compared (modified plaque index kappa = 0.90, probing depth kappa = 0.90, probing bleeding index kappa = 1).

### 2.4. Statistical Analysis

The component ratio was used to describe the proportion of each indicator in the control group and the case group. All indicators in this study were categorical variables, univariate Logistic regression was used to analyze the correlation between each indicator and the occurrence of peri-implant disease. Multivariate Logistic regression was used to comprehensively analyze the influence of univariate factors with statistical significance (*p*-values < 0.05). The odds ratio (OR) and its 95% confidence interval (95% CI) were used to represent the strength of association between risk factors and peri-implant disease. All statistical analyses were carried out by SAS 9.4 software programming, and the test level was α = 0.05.

## 3. Results

Finally, 131 participants (248 implants) were enrolled, the examined population included 58 men and 73 women with a mean age of 48.29 ± 11.85 years. The mean observation time since implant placement was 2.52 years. 68 patients (110 implants) developed the peri-implant disease after the mean functional time of 1.85 ± 0.70 years. The peri-implant disease prevalence at implant level was 44.35% (95% CI: 38.18–50.53%), and the prevalence rate at patient level was 51.91% (95% CI: 43.35–60.47%). The prevalence of peri-implant mucositis and peri-implantitis was 45.80% (95% CI: 37.27–54.33%) and 7.63% (95% CI: 3.08–12.18%) at the subject level, and 36.69% (95% CI: 30.69–42.69%) and 7.66% (95% CI: 4.35–10.97%) at the implant level.

Single-factor analysis exhibited that male sex, bleeding on brushing, periodontitis caused tooth loss at the implant site, tooth brushing once a day, moderate periodontitis, implant length < 10mm, poor proximal contact of the prosthesis, demonstrated statistical significance. The data of single-factor analysis were shown in Table 1 and Table 2.

For factors that were statistically significant in univariate analysis, multivariate analysis was used, which revealed that male [OR = 1.91; 95% CI: 1.02–3.57; *p* = 0.04], implant length < 10 mm (OR = 7.87; 95% CI: 1.62–38.46; *p* = 0.01), poor proximal contact of the prosthesis (OR =1.90; 95% CI: 1.06–3.42; *p* = 0.03), tooth brushing once a day (OR = 3.11; 95% CI: 1.26–7.68; *p* = 0.04) and moderate periodontitis (OR = 13.00; 95% CI: 4.38–38.60; *p* < 0.01) were independent risk factors for peri-implant disease. The data were shown in Table 3.

## 4. Discussion

Implant therapy had become widely accepted as a method of oral rehabilitation in partially edentulous patients, this study evaluated the prevalence and risk factors of peri-implant diseases in a population of western China partially edentulous individuals rehabilitated with implant-supported prostheses. More than 50% of the participants had peri-implant disease, indicating that peri-implant diseases were remarkably frequent in patients with dental implants. Male sex, implant length < 10 mm, poor proximal contact of the prosthesis, tooth brushing once a day and moderate periodontitis were identified as risk indicators of peri-implant disease. Such a high prevalence occurred in the early stages of implantation, suggesting that we should pay attention to the occurrence of peri-implant diseases and control the risk factors in the initial phases of implantation.

In the present study, the prevalence of peri-implant disease was 44.35% on the implant level, of which the prevalence of peri-implant mucositis was 36.69% and peri-implantitis was 7.66%. The meta-analysis of Lee et al. [14] included 47 studies with an average follow-up period of more than 3 years, and concluded a weighted mean prevalence on the implant level of 46.83% (95% CI: 38.30–55.36%) of peri-implant mucositis, and 9.25% (95% CI: 7.57–10.93%) of peri-implantitis, Lee et al. considered the functional time was associated with the prevalence of peri-implantitis. The prevalence of peri-implant mucositis on the implant level in the present study was similar to the findings of Lee et al., while the prevalence of peri-implant mucositis was lower than level of 9.25%, which may be related to the shorter functional time. Romandini et al. [15] reported a prevalence of peri-implant mucositis and peri-implantitis respectively 31.9% and 31.7% at implant level, while Matarazzo et al. [16] found 69.2% and 20.5%, respectively. The difference in the prevalence results between these and the present study could be due to the varied case defining criteria applied. While Romandini et al. [15] defined peri-implantitis in the presence of BOP/SOP together with radiographic bone loss ≥2 mm, Matarazzo et al. [16] considered the presence of BOP and suppuration, along with marginal bone level ≥3 mm as the threshold for peri-implantitis.

Of the 24 factors included, 7 factors exhibited statistical significance in univariate analysis. Among them, 5 factors were related to the patient characteristics, such as male sex, bleeding on brushing, periodontitis caused tooth loss at the implant site, tooth brushing once a day and moderate periodontitis, whereas 2 factors were related to oral hygiene maintenance such as implant length < 10 mm and poor proximal contact of the prosthesis. After multivariate analysis, bleeding on brushing and periodontitis caused tooth loss at the implant site no longer had statistical significance, possibly because their effect was modest compared to other factors, and these two factors were part of periodontitis’ clinical manifestations as well.

Periodontitis was a chronic multifactorial inflammatory disease associated with plaque biofilms characterized by progressive destruction of the tooth-supporting apparatus. In this investigation, periodontitis was found to play a critical role in peri-implant disease, the Logistic regression for multiple factors analysis exhibited that the risk of peri-implant disease in patients with moderate periodontitis was 13 times that in healthy people. Periodontitis was considered a risk factor for peri-implantitis in the consensus report of the 2017 World Workshop [9], similarly, researchers discovered that having a history of periodontitis was linked to a lower survival rate and higher risk of peri-implantitis during a 5–10 years period after implant loading in a meta-analysis [17]. Smith et al. [18] suggested that long-term management of patients with periodontitis should pay particular attention on smoking cessation, plaque control, prosthetic issues affecting plaque removal and supportive peri-implant therapy. All of these pointed to the need of controlling patients’ periodontitis prior to implantation, and dentists must convince patients to pay close attention to their periodontal health. However, some researchers discovered that individuals with periodontitis maintained peri-implant health after implantation, implying that, in addition to tight periodontitis control before surgery, regular follow-up and plaque control following implantation were critical.

Plaque biofilm in the oral cavity was closely related to periodontal and peri-implant disease [9,19], as a means of daily oral maintenance, brushing teeth was of great significance for reducing plaque. The present study found that patients who brushed their teeth only once a day (poor brushing habits) had a 3 times higher risk of peri-implant disease than those who brushed their teeth every morning and evening (brushing twice a day), suggesting that regular supportive maintenance after implant placement was critical for maintaining implant health. For partially edentulous patients, the remaining teeth were the primary source of bacteria for implant colonization, thus, plaque management was critical for the long-term health of implants. According to our investigation, up to 85.89% (213/248) of the survey population had never scaling after implantation. It must be given sufficient attention that both professional oral maintenance and patient self-maintenance were important for peri-implant health. Dentists should educate patients about the necessity of maintaining good oral hygiene and guide patients’ maintenance of oral hygiene correctly and effectively, at the same time, prostheses should be designed to be accessible for self-cleaning and brushing.

Male patients had 1.9 times higher risk of peri-implant disease than female patients in this study, and men were more prone to peri-implant disease than women. The proportion of male patients with periodontitis was higher than that of female patients in this study (Table 3), and the difference was statistically significant (*p* < 0.05), which could be one of the causes for the above results.

Implant related factors including implant length < 10 mm (OR = 7.87; 95% CI: 1.62–38.46; *p* = 0.01) and poor proximal contact of the prosthesis (OR = 1.90; 95% CI: 1.06–3.42; *p* = 0.03) were risk factors for developing peri-implant disease.

Inadequate bone height and the presence of anatomical structures such as a low located maxillary sinus made it not always possible to place a dental implant of a certain length. However, implant length < 10 mm (OR = 7.87; 95% CI: 1.62–38.46; *p* = 0.01) had 7.87 times higher risk of peri-implant disease than implant length ≥ 10 mm in this study. According to a meta-analysis, short implants showed a 2.5 times higher risk of failure than long implants [20], which was consistent with our findings. As shown above, the preoperative implant selection would also have a significant effect on peri-implant health, and dentists should be aware of this. Short dental implants were still an option for implant placement owing to a couple of conditions; nevertheless, a proper treatment plan should be considered; long (≥10 mm) dental implants might be chosen if the conditions permit.

Optimizing the proximal contact was important to prevent tooth displacement, food impaction, recurrent tooth caries, and periodontal disease [21], as for the present investigation, the risk of peri-implant disease was 1.90 times higher for implants with poor proximal contact than for implants with good proximal contact. Several factors, including implant functional years, frequent use of interdental brushes, food impaction, a good proximal contact relationship was not established during the restoration might associate with the poor proximal contact [22]. The vertical movement distance of implant was much smaller than that of natural teeth during mastication, so a gap could be formed between the proximal teeth and the implant, which was one of the most widely accepted assumptions for mesial proximal contact loss. Once the proximal contact was lost, the implant would become the end of force transfer, and the force couldn’t be effectively dispersed, which would increase the force exerted on the implant itself and increase the risk of complications. Poor proximal contact would exacerbate inflammation caused by plaque biofilms, eventually leading to bone loss [23], so it was critical for the dentists to appropriately restore the proximal contact while rehabilitation.

One of the limitations of the present study was that patients needed to recall the pervious oral condition while filling the questionnaire, which might be different from the actual situation, like bleeding on brushing and causes of tooth loss at the implant site. Additionally, patient’s previous oral status, like periodontal condition, were required to establish a better baseline. Since certain medical records did not indicate the periodontal condition, the degree of periodontitis in this study was examined uniformly over the follow-up to ensure consistency, which may differ from the periodontal condition before implantation. Therefore, future prospective studies were required to address the issue.

## 5. Conclusions

Taking into account the limitations of the present study, the findings revealed that the prevalence and risk factors of the peri-implant diseases in the studied population of western China were not significantly different from those reported elsewhere. Peri-implant diseases had affected 51.91% of the studied population, reinforcing the perception that patients must be made aware of the risk of peri-implant diseases. Dentists should conduct a thorough examination of patients before implantation, inform patients to control periodontitis and other risk factors, and guide patients to maintain oral hygiene habits. When developing the implant scheme, extra attention should be made to the selection of long implants and proximal contact of the prosthesis designs.

## Figures and Tables

**Table 1:** Univariate Logistic regression analysis of patient-related factors.

Variables	Control Group (*n* = 138, %)	Case Group (*n* = 110, %)	χ^2^	*P*	OR (95% CI)
Gender					
Male	54 (39.13)	61 (55.45)	6.06	0.01 *	1.89 (1.14–3.14)
Female	84 (60.87)	49 (44.55)			1
Age					
<35	25 (18.12)	14 (12.73)			1
35–65	108 (78.26)	90 (81.82)	0.002	0.97	1.49 (0.73–3.03)
>65	5 (3.62)	6 (5.45)	0.79	0.37	2.14 (0.55–8.31)
Smoking					
Never	109 (78.99)	83 (75.46)			1
Smoking	22 (15.94)	21 (19.09)	0.16	0.69	1.25 (0.65–2.43)
Given up	7 (5.07)	6 (5.45)	0.0001	0.99	1.13 (0.37–3.48)
Drinking					
No	105 (76.09)	86 (78.18)			1
Moderate	26 (18.84)	18 (16.36)	0.21	0.65	0.85 (0.44–1.64)
Heavy	7 (5.07)	6 (5.45)	0.05	0.82	1.05 (0.34–3.23)
Diabetes					
Yes	15 (10.87)	13 (11.82)	0.06	0.81	1.10 (0.50–2.42)
No	123 (89.13)	97 (88.18)			1
Cardiovascular disease					
Yes	36 (26.09)	34 (30.91)	0.70	0.40	1.27 (0.72–2.21)
No	102 (73.91)	76 (69.09)			1
Osteoporosis					
Yes	4 (2.9)	6 (5.45)	1.00	0.32	1.93 (0.53–7.03)
No	134 (97.1)	104 (94.55)			1
Bleeding on brushing					
Yes	41 (29.71)	51 (46.36)	7.18	0.01 *	2.05 (1.21–3.45)
No	97 (70.29)	59 (53.64)			1
Cause of tooth loss at implant site					
Trauma	29 (21.01)	12 (10.91)			1
Decay	79 (57.25)	60 (54.55)	0.05	0.82	1.84 (0.87–3.89)
Periodontitis	13 (9.42)	28 (25.45)	12.06	0.0005^*^	5.20 (2.03–13.33)
Others	17 (12.32)	10 (9.09)	0.86	0.36	1.42 (0.51–3.98)
Count of scaling after implantation					
0	116 (84.06)	97 (88.18)	0.23	0.63	0.84 (0.20–3.43)
1	15 (10.87)	7 (6.36)	0.89	0.34	0.47 (0.09–2.43)
2	4 (2.9)	4 (3.64)			1
3	3 (2.17)	2 (1.82)	0.01	0.92	0.67 (0.07–6.41)
Frequency of brushing per day					
1	11 (7.97)	24 (21.82)	5.42	0.02^*^	3.54 (1.63–7.68)
2	112 (81.16)	69 (62.73)			1
≥3	15 (10.87)	17 (15.45)	0.003	0.96	1.84 (1.86–3.92)
Gargle					
Yes	89 (64.49)	61 (55.45)			1
No	49 (35.51)	49 (44.55)	2.09	0.15	1.46 (0.87–2.44)
DIS					
No	12 (8.70)	8 (7.27)			1
<1/3	108 (78.26)	73 (66.36)	0.0005	0.98	1.01 (0.40–2.60)
≥1/3&≤2/3	16 (11.59)	29 (26.36)	0.0009	0.98	2.72 (0.92–8.03)
>2/3	2 (1.45)	0 (0.00)	0.0006	0.98	<0.01 (<0.01–>999.99)
CIS					
No	13 (9.42)	2 (1.82)			1
<1/3	72 (52.17)	63 (52.27)	3.28	0.07	5.68 (1.24–26.15)
≥1/3&≤2/3	50 (36.23)	44 (40.00)	3.18	0.07	5.72 (1.22–26.73)
>2/3	3 (2.17)	1 (0.91)	0.11	0.74	2.17 (0.14–32.50)
Periodontitis degree					
No	45 (32.61)	7 (6.36)			1
Slight	79 (57.25)	56 (50.91)	0.004	0.95	4.56 (1.92–10.84)
Moderate	14 (10.14)	47 (42.73)	37.80	<0.01 *	21.58 (7.98–58.38)

* *p* < 0.05.

**Table 2:** Univariate Logistic regression analysis of implant-related factors.

Variables	Control Group (*n* = 138, %)	Case Group (*n* = 110, %)	χ^2^	*P*	OR (95% CI)
Implant system					
Nobel	56 (44.44)	50 (45.87)			1
Biomet 3i	49 (38.89)	45 (41.28)	1.59	0.21	1.03 (0.59–1.79)
ITI	16 (12.70)	13 (11.93)	0.55	0.46	0.91 (0.40–2.08)
Others	5 (3.97)	1 (0.92)	1.77	0.18	0.22 (0.03–1.98)
Implant length					
<10 mm	14 (10.14)	2 (1.82)	5.55	0.02 *	6.10 (1.36–27.43)
≥10 mm	124 (89.86)	108 (98.18)			1
Transgingival					
Yes	114 (82.61)	92 (83.64)	0.05	0.83	0.93 (0.48–1.82)
No	24 (17.39)	18 (16.36)			1
Alveolar bone type					
II	60 (43.48)	45 (40.91)			1
III	77 (55.80)	61 (55.45)	1.74	0.19	1.06 (0.63–1.76)
IV	1 (0.72)	4 (3.64)	2.14	0.14	5.33 (0.57–49.35)
Bone graft					
No	52 (37.68)	47 (42.73)			1
Yes	86 (62.32)	63 (57.27)	0.65	0.42	0.81 (0.49–1.35)
Immediate implant					
No	110 (79.71)	92 (83.64)			1
Yes	28 (20.29)	18 (16.36)	0.62	0.43	0.77 (0.40–1.48)
Prosthetic					
Single crown	66 (47.83)	58 (52.73)			1
Splinted prosthetic	59 (42.75)	39 (35.45)	1.37	0.24	0.75 (0.44–1.29)
Fixed partial denture	13 (9.42)	13 (11.82)	0.43	0.51	1.14 (0.49–2.65)
Implant-abutment connection					
Cement retention	125 (90.58)	98 (89.09)			1
Screw retention	13 (9.42)	12 (10.91)	0.15	0.70	1.18 (0.51–2.70)
Proximal contact					
Good	103 (74.64)	68 (61.82)			1
Poor	35 (25.36)	42 (38.18)	4.65	0.03 *	1.82 (1.06–3.13)
Opposite jaw type					
Tooth	114 (82.61)	78 (70.90)			1
Mental crown	5 (3.62)	9 (8.18)	1.28	0.26	2.63 (0.85–8.15)
Porcelain crown	7 (5.07)	15 (13.64)	2.95	0.09	3.13 (1.22–8.04)
Metal-porcelain crown	6 (4.35)	4 (3.64)	0.63	0.48	0.97 (0.27–3.57)
Porcelain fixed partial denture	6 (4.35)	4 (3.64)	0.63	0.48	0.97 (0.27–3.57)

* *p* < 0.05.

**Table 3:** Multiple Logistic regression analysis of 248 implants.

Variables	χ^2^	*P*	OR (95% CI)	OR (95% CI)
Gender				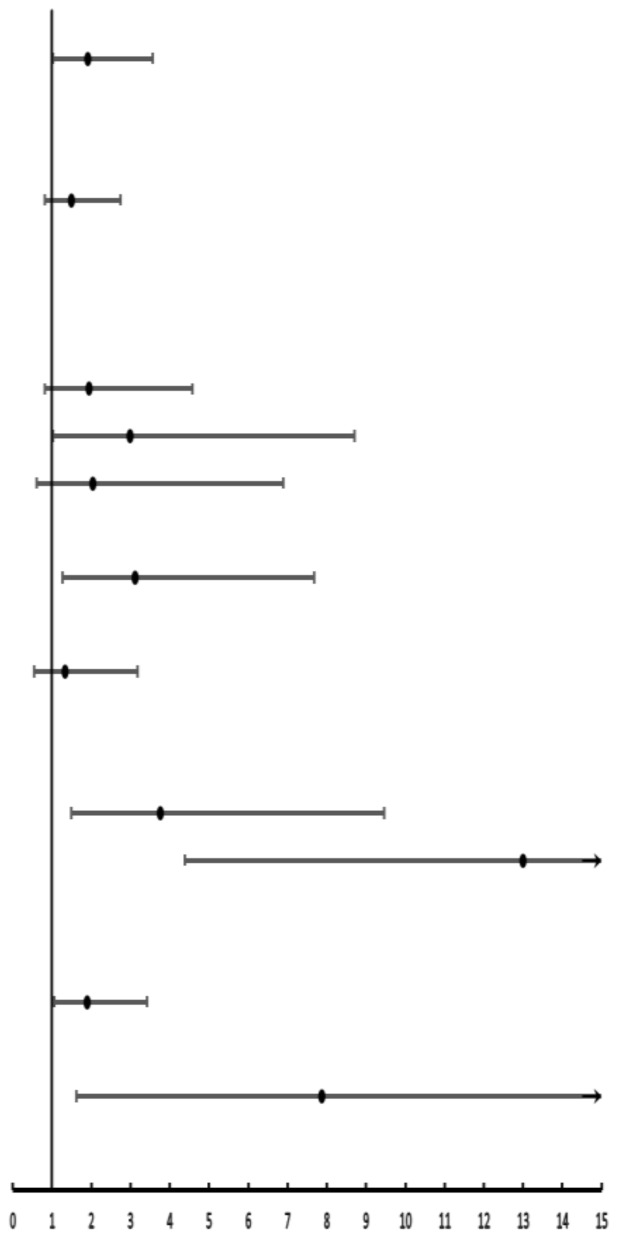
Male	4.10	0.04 *	1.91 (1.02–3.57)	
Female			1	
Bleeding on brushing				
Yes	1.65	0.20	1.49 (0.81–2.74)	
No			1	
Cause of tooth loss at implant site				
Trauma			1	
Decay	0.04	0.85	1.94 (0.82–4.58)	
Periodontitis	2.18	0.14	2.99 (1.03–8.71)	
Others	0.05	0.82	2.03 (0.60–6.89)	
Frequency of brushing per day				
1	4.21	0.04 *	3.11 (1.26–7.68)	
2			1	
≥3	0.36	0.55	1.33 (0.55–3.18)	
Periodontitis degree				
No			1	
Slight	0.02	0.90	3.76 (1.49–9.46)	
Moderate	21.28	<0.0001 *	13.00 (4.38–38.60)	
Proximal contact				
Good			1	
Poor	4.66	0.03 *	1.90 (1.06–3.42)	
Implant length				
<10 mm	6.55	0.01 *	7.87 (1.62–38.46)	
≥10 mm			1	
			
			
			
			
			
			

* *p* < 0.05.

## Data Availability

The data presented in this study are available are available from the corresponding author on reasonable request.

## References

[1] Berglundh T., Armitage G., Araujo M.G., Avila-Ortiz G., Blanco J., Camargo P.M., Chen S., Cochran D., Derks J., Figuero E. (2018). Peri-Implant Diseases and Conditions: Consensus Report of Workgroup 4 of the 2017 World Workshop on the Classification of Periodontal and Peri-Implant Diseases and Conditions. J. Clin. Periodontol..

[2] Renvert S., Persson G.R., Pirih F.Q., Camargo P.M. (2018). Peri-Implant Health, Peri-Implant Mucositis, and Peri-Implantitis: Case Definitions and Diagnostic Considerations: Diagnostic Criteria of Peri-Implant Health and Diseases. J. Periodontol..

[3] Derks J., Tomasi C. (2015). Peri-Implant Health and Disease. A Systematic Review of Current Epidemiology. J. Clin. Periodontol..

[4] Kordbacheh Changi K., Finkelstein J., Papapanou P.N. (2019). Peri-Implantitis Prevalence, Incidence Rate, and Risk Factors: A Study of Electronic Health Records at a U.S. Dental School. Clin. Oral Implant. Res..

[5] Monje A., Wang H.-L., Nart J. (2017). Association of Preventive Maintenance Therapy Compliance and Peri-Implant Diseases: A Cross-Sectional Study. J. Periodontol..

[6] Rokn A., Aslroosta H., Akbari S., Najafi H., Zayeri F., Hashemi K. (2017). Prevalence of Peri-Implantitis in Patients Not Participating in Well-Designed Supportive Periodontal Treatments: A Cross-Sectional Study. Clin. Oral Implant. Res..

[7] French D., Grandin H.M., Ofec R. (2019). Retrospective Cohort Study of 4591 Dental Implants: Analysis of Risk Indicators for Bone Loss and Prevalence of Peri-implant Mucositis and Peri-implantitis. J. Periodontol..

[8] Derks J., Schaller D., Håkansson J., Wennström J.L., Tomasi C., Berglundh T. (2016). Effectiveness of Implant Therapy Analyzed in a Swedish Population: Prevalence of Peri-Implantitis. J. Dent. Res..

[9] Schwarz F., Derks J., Monje A., Wang H.-L. (2018). Peri-Implantitis. J. Periodontol..

[10] Heitz-Mayfield L.J.A., Salvi G.E. (2018). Peri-Implant Mucositis. J. Clin. Periodontol..

[11] Armitage G.C. (1999). Development of a Classification System for Periodontal Diseases and Conditions. Ann. Periodontol..

[12] Sanz M., Chapple I.L., Working Group 4 of the VIII European Workshop on Periodontology (2012). Clinical Research on Peri-Implant Diseases: Consensus Report of Working Group 4. J. Clin. Periodontol..

[13] Caton J.G., Armitage G., Berglundh T., Chapple I.L.C., Jepsen S., Kornman K.S., Mealey B.L., Papapanou P.N., Sanz M., Tonetti M.S. (2018). A New Classification Scheme for Periodontal and Peri-Implant Diseases and Conditions - Introduction and Key Changes from the 1999 Classification. J. Clin. Periodontol..

[14] Lee C.-T., Huang Y.-W., Zhu L., Weltman R. (2017). Prevalences of Peri-Implantitis and Peri-Implant Mucositis: Systematic Review and Meta-Analysis. J. Dent..

[15] Romandini M., Lima C., Pedrinaci I., Araoz A., Soldini M.C., Sanz M. (2021). Prevalence and Risk/Protective Indicators of Peri-Implant Diseases: A University-Representative Cross-Sectional Study. Clin. Oral Implant. Res..

[16] Matarazzo F., Sabóia-Gomes R., Alves B.E.S., de Oliveira R.P., Araújo M.G. (2018). Prevalence, Extent and Severity of Peri-Implant Diseases. A Cross-Sectional Study Based on a University Setting in Brazil. J. Periodont. Res..

[17] Carra M.C., Rangé H., Swerts P.-J., Tuand K., Vandamme K., Bouchard P. (2022). Effectiveness of Implant-Supported Fixed Partial Denture in Patients with History of Periodontitis: A Systematic Review and Meta-Analysis. J. Clin. Periodontol..

[18] Smith M.M., Knight E.T., Al-Harthi L., Leichter J.W. (2017). Chronic Periodontitis and Implant Dentistry. Periodontology 2000.

[19] Lamont R.J., Koo H., Hajishengallis G. (2018). The Oral Microbiota: Dynamic Communities and Host Interactions. Nat. Rev. Microbiol..

[20] Abdel-Halim M., Issa D., Chrcanovic B.R. (2021). The Impact of Dental Implant Length on Failure Rates: A Systematic Review and Meta-Analysis. Materials.

[21] Liang C., Nien C., Chen Y., Hsu K. (2020). The Prevalence and Associated Factors of Proximal Contact Loss between Implant Restoration and Adjacent Tooth after Function: A Retrospective Study. Clin. Implant. Dent. Relat. Res..

[22] Bento V.A.A., Gomes J.M.L., Lemos C.A.A., Limirio J.P.J.O., Rosa C.D.D.R.D., Pellizzer E.P. (2021). Prevalence of Proximal Contact Loss between Implant-Supported Prostheses and Adjacent Natural Teeth: A Systematic Review and Meta-Analysis. J. Prosthet. Dent..

[23] De Kok I.J., Duqum I.S., Katz L.H., Cooper L.F. (2019). Management of Implant/Prosthodontic Complications. Dent. Clin. North Am..

